# Acute administration of Manning compound during the spawning period reduces reproductive success in female zebrafish

**DOI:** 10.3389/fendo.2026.1771879

**Published:** 2026-04-30

**Authors:** Dinusha Rajapaksha, Taanishi Wadhi, Marina Spitz, Varshika Segar, Niepukolie Nipu, Vance L. Trudeau, Jan A. Mennigen

**Affiliations:** Department of Biology, University of Ottawa, Ottawa, ON, Canada

**Keywords:** nonapeptides, receptors, reproduction, vasopressin, vasotocin, prostaglandin, courtship behaviour

## Abstract

**Introduction:**

The vertebrate nonapeptide vasotocin (Avp) is evolutionarily conserved and homologous to mammalian vasopressin. Previous work using *avp*^-/-^ zebrafish demonstrated a role for Avp in female, but not male, zebrafish reproduction. The phenotype manifested as reductions in pair-breeding success, viable egg release, ovarian Prostaglandin F_2a_ (PGF_2a_) concentration, and reduction in ovarian transcripts coding for PGF_2a_. synthesis enzymes. However, mutants exhibited life-long hypercortisolism, confounding the investigation of the mechanistic basis of Avp in female zebrafish reproductive success.

**Methods:**

Here, we used acute pharmacological manipulation via the Avpr1 receptor antagonist Manning compound alone (dose-response experiment) or in conjunction with PGF_2a_ (rescue experiment) to investigate the role of Avp1-type receptor (Avpr1) signalling in female zebrafish reproductive success.

**Results:**

In the dose-response experiment, acute female intraperitoneal injection of MC at the lowest dose tested (5 ng/g bodyweight) reduced breeding pair success, quivering behaviour, and the number of released viable eggs compared to saline-injected controls. Whole-body cortisol and reproductive hormone concentrations, clutch size, hatchability and larval survival were unaffected at this concentration. Additional effects on courtship behaviours and increases in estradiol and progesterone concentrations were observed exclusively in females injected with higher doses of MC, suggesting no contribution to the reproductive phenotype observed at the lowest MC dose. The rescue experiment revealed that the phenotype elicited by 5 ng/g bw MC was rescued by co-injection with 5 mg/g bw PGF_2a_.

**Discussion:**

Together, these data suggest that Avp acutely promotes female zebrafish spawning through action on Avpr1 receptors and likely involve downstream activation of PGF_2a_. Because Avp may regulate female reproductive success at the ovarian level, future studies should explore the location, regulation, and function of the ovarian Avp receptors in detail.

## 1 Introduction

The nonapeptide vasotocin is the teleost homologue of mammalian vasopressin and is conserved in vertebrate evolution (1). In zebrafish, vasotocin (*avp* in ZFIN (www.zfin.org) nomenclature) is expressed in neurons of the preoptic area, from where fibres extend to the pituitary and released into the bloodstream as a hormone (2). Avp neurons also extend to extrahypothalamic regions linked to integration of reproductive cues and mediation of courtship behaviours, highlighting its potential role as a neuromodulator (2). In addition to brain and pituitary, Avp is expressed in the ovary (3), and may thus modulate hypothalamus-pituitary-gonadal (HPG) axis function at multiple levels. In zebrafish, Avp signalling involves five Avp receptors (AvpRs) encoded in the genome. These can be divided into *avpr1* receptor paralogues (*avpr1aa*, *avpr1ab*) and *avpr2* receptor paralogues (*avpr2aa*, *avpr2ab*), and homologues (*avpr2ba, avpr2l)* (4, 5).

In teleost fishes, reproductive roles for Avp have been described (4). Using *avp*^-/-^ mutants, a role for Avp in female, but not male, zebrafish reproduction was identified, with a reproductive phenotype characterized by reduced numbers of total and viable eggs, decreased ovarian PGF_2α_ concentration and transcripts coding for enzymes of PGF_2α_ synthesis from arachidonic acid, and decreased quivering behaviour linked to egg release (3). While these findings suggest a role for ovarian action of Avp to stimulate ovarian PGF_2α_ release in line with reported effects in Asian stinging catfish (Heretopneustes fossilis). oocytes *in vitro* (6), they reveal the importance of the Avp system on female spawning in a genetically tractable fish model *in vivo.* Previous work using Avp and AvpR knock-outs in medaka (*Oryzias latipes*) had focused, in line with the historically separate investigation of Avp’s neuromodulatory and modulatory function of the endocrine HPG axis in fishes (4), exclusively on male courtship behaviour, reporting a role for Avp and Avpr1 in male mate-guarding behaviour in this species (Yokoi et al., 2015).

The findings that Avp plays a role in female zebrafish spawning (3) also point to the likely wider evolutionary conservation and importance of this mechanism among Otophysi and/or teleost fishes in general, beginning to address an identified need for comparative study of reproductive roles of the Avp system in diverse physiology of female fish reproduction (6). However, and perhaps unsurprisingly given the pleiotropic role of Avp in fishes (7), *avp*^-/-^ mutants also exhibited life-long hypercortisolism, which may secondarily have affected the female reproductive phenotype previously reported (3). By using acute pharmacological approaches, we here test the hypothesis that (i) Avp acutely promotes reproductive success in wildtype female zebrafish with normal ontogenesis of the Avp system *in vivo* via G-protein coupled Avpr1 receptors, and that (ii) this effect is dependent on downstream activation of ovarian PGF_2α_ release and function. We predict that Manning compound (MC), an Avpr1 receptor antagonist active in fish (8, 9), will reduce female ovulation similar to *avp*^-/-^ mutants (3), and that concurrent injection of PGF_2α_ will rescue the observed reduction in ovulation.

## 2 Materials and methods

### 2.1 Animals

Sexually mature, 6-11 months old wild-type male and female zebrafish (AB strain) were obtained from in-house breeding stock at the University of Ottawa. Fish were maintained in a recirculating system (Techniplast, Montréal, QC, Canada) housed in 12 L tanks at a density of 3 fish/L. The system used reverse osmosis (RO) water supplemented with marine salts (Instant Ocean, Blacksburg, VA, USA), maintained at a pH of 7.3, a conductivity of 400 µS, and a temperature of 28 °C, under a 14:10 h light–dark cycle. Upon the onset of exogenous feeding, larvae were fed twice daily with appropriately sized GEMMA diets (Skretting, Vancouver, BC, Canada). Adult zebrafish received a mixed diet twice daily (Adult Zebrafish diet, Zeigler Bros Inc, Gardners, PA, USA; Larval AP-100, Zeigler Bros Inc, Gardners, PA, USA; Golden Pearls, Artemia International, Fairview, TX, USA). Two weeks before the experiment, sexually mature fish were separated by sex. Separation by sex was chosen to harmonize reproductive physiology baseline, as it avoids the confound that some, but not all fish in mixed-tank set-ups may engage in mating and or group spawning events prior to experimentation. All procedures were conducted under the guidelines of the Canadian Council on Animal Care and approved by the University of Ottawa Animal Care Protocol Review Committee (Protocol #BL-4511).

### 2.2 Breeding assays and pharmacological targeting of Avpr1 receptors

**Figure 1:**
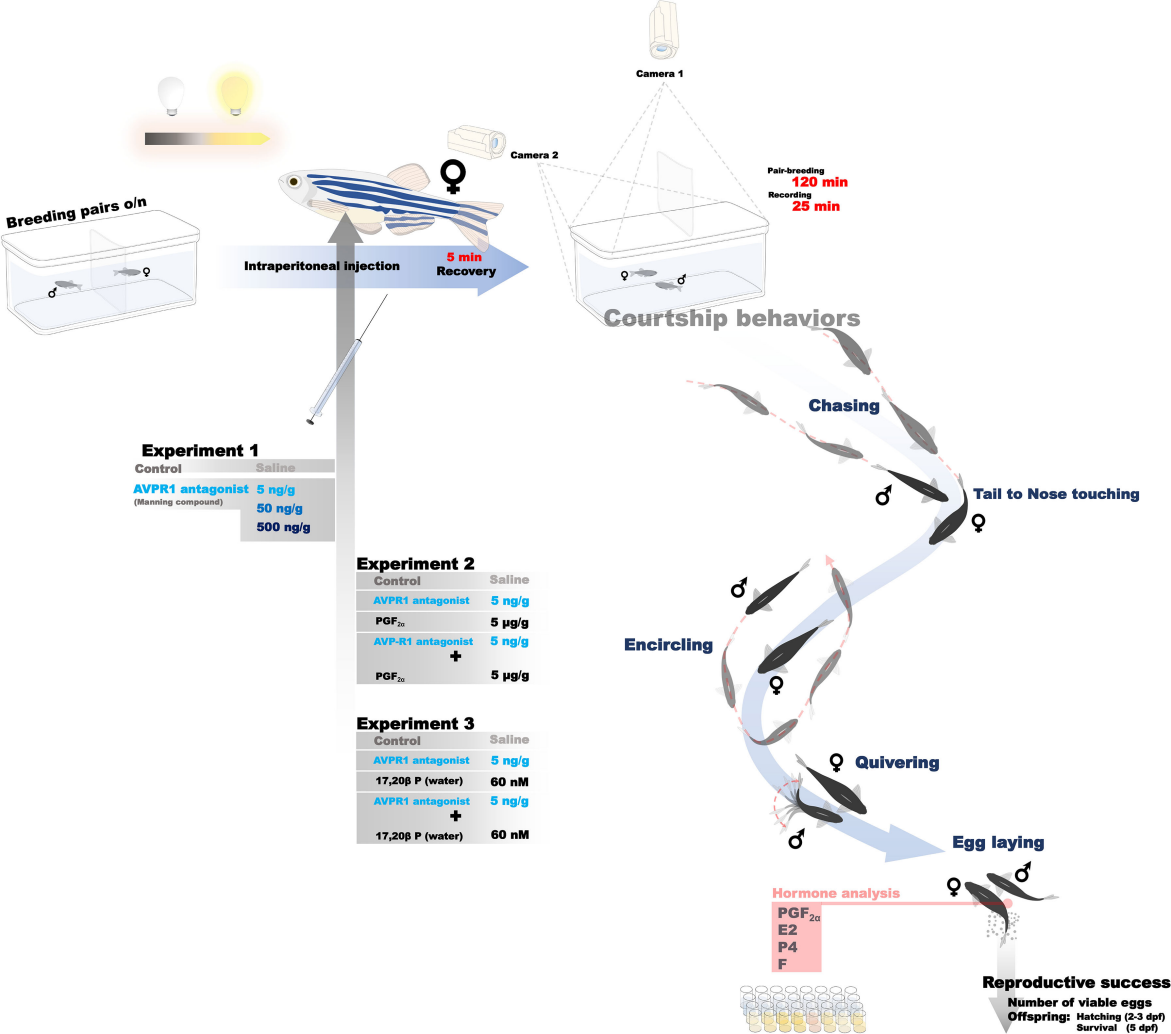
Overview of experimental design to establish dose-response relationships between acute injection of MC in females and indices of their reproductive success, female whole body reproductive hormone levels, and courtship behaviour in breeding pairs. Rescue experiments consisting of co-injection of 5 ng/g MC and reproductive hormones followed the same design with the exception of hormone analyses.

To determine the Avpr1-dependency of female reproductive indices previously reported in *avp*^-/-^ KO zebrafish (3), we used acute intraperitoneal (i.p.) injection of physiological saline (control) and the selective vasopressin 1A receptor antagonist (d(CH_2_)_5_^1^, Tyr(Me)^2^, Arg^8^) vasopressin (Tocris Bio-Techne, Oakville, ON, Canada), also termed Manning Compound (MC). MC has previously been shown to be active and specific in fishes in general (10), and zebrafish in particular (2, 11). Regarding MC affinities for nonapeptide receptors in fishes, a study expressing a white sucker (*C. commersonii)* Avpr1-type receptor in a heterologous system (10) demonstrated that (i) Avpr1-type receptor was highly selective for Avp over isotocin, the teleost oxytocin-family peptide (>1000-fold), and that (ii) a 5-fold molar excess of MC was efficient in antagonizing Avp action on AvpR1-type receptor signaling in this system. While the oxytocin receptor (Oxtr) in the same species was found to be much less discriminatory between isotocin compared to Avp, its affinity to MC was unfortunately not formally investigated (12). As such, it is unknown whether MC may also weakly antagonize Oxtr in teleosts, as has been suggested for some specific *in vitro* studies in the rat (9). Initial experiments were conducted using 6–11-month-old sexually mature zebrafish to establish an effective dose range for MC (**Figure 1**). Following anaesthesia using 0.24 mg/mL tricaine (Syndel Laboratories), female zebrafish were i.p. injected with either physiological saline (n=43), 5 ng/g MC (n=31), 50 ng/g MC (n=35) or 500 ng/g MC (n=29). In all cases, a standardized injection volume of 10 μL/g bw was used at 8:30 am (lights on). Following i.p. injection, female fish were allowed to recover for 5 min. The MC dose ranges, route of administration, and recovery times are in line with previous work in teleost fishes (2, 11, 13–15) and other species (16, 17). After the recovery period, females were re-introduced male fish in the breeding chamber which had been set-up the previous day for the pair, and in which male and female had been separated by a translucent Plexi-glass barrier. The Plexi-glass barrier was then removed, and fish allowed to breed for a period of 2 h. After the breeding period, reproductive success was assessed by quantifying the percentage of breeding pairs that produced fertilized eggs, and counting the number of viable fertilized eggs per pair. To assess possible role for oxytocin receptors (Oxtr) in affecting these measures of reproductive success, a supplementary experiment using i.p. saline injection control (n=20), and two doses of the Oxtr antagonist L-368,899 (50 ng/g and 500 ng/g, n=20 each), whose specificity for Oxtra and Oxtrb receptors over AvpR1 receptors had been validated in zebrafish (18), was conducted. In a subset of breeding pair egg batches (n=4-16, depending on the number of successful breeding pairs in treatment groups) obtained from the saline and MC groups (Experiment 1), key developmental milestones of hatching and larval survival were determined at 2–3 days post-fertilization (dpf) and 5 dpf, respectively. To determine the number of fertilized eggs, all spawned eggs were collected from the breeding chamber inlets and transferred to Petri dishes containing standard E3 medium. Fertilized eggs were identified as translucent and symmetrical with an expanded perivitelline space and counted. Unfertilized eggs (yellow, granular) and dead eggs (white, degraded) were recorded separately and removed. Hatching rate and larval survival were determined by assessing the number of hatched larvae at 2–3 dpf as a percentage of total fertilized eggs in a given batch, and by assessing larval survival at 5 dpf as a percentage of total fertilized eggs in a given batch, respectively.

A subset of breeding pairs (n=17-19 per treatment group) were recorded for the first 25 min using a set-up allowing for the simultaneous filming of four tanks at a time. While each treatment group (i.p. injection of physiological saline, 5 ng/g MC, 50 ng/g MC, 500 ng/g MC) was included for each recording, positioning of treatment group tanks was randomized. The time of recording time was based on previous work in our laboratory revealing that courtship behaviour decreases over the entire 2 h time-period in breeding pairs. In all cases, four individual cameras were positioned at the side of each tank and one camera on top of each tank, allowing to capture both lateral and top view videos for all investigated pairs. Recordings were captured with VIXIA HF R800 camcorders (Canon Canada, Brampton, ON, Canada) with a resolution of 1080p at 60 frames/s. Females from another subset of breeding pairs (n=4-7 per treatment group) were euthanized by rapid cooling and spinal transection before being flash frozen for whole body steroid extraction and hormone quantification analyses.

### 2.3 Rescue experiments

#### 2.3.1 Prostaglandin F_2α_ rescue experiment

Full-factorial design rescue experiments were performed to evaluate whether prostaglandin F_2α_ (PGF_2α_; Sigma-Aldrich, Oakville, ON, Canada), which had been found to be significantly reduced in female *avp*^-/-^ zebrafish (3) could restore reproductive outcomes impaired by 5 ng/g MC injection following the same experimental procedure as above (**Figure 1**). Using the pair-breeding set-up described above, four treatment groups (n=21-24) were investigated: Females i.p. injected with physiological saline, females i.p. injected with 5 ng/g bwMC, females injected with PGF_2α_ 5 μg/g bw (19), and females injected with a combination of 5 ng/g bw MC and 5 μg/g bw PGF_2α_. Following i.p injections and a 5-min recovery period, the divider was removed at the onset of the light cycle (8.30 h) and breeding pairs were allowed to directly interact and spawn for a period of 2 h. Indices of reproductive success were analysed, and courtship behaviours were analysed for the first 25 min in a subset (n=15-16 per treatment group), as previously described.

#### 2.3.2 Maturation-inducing steroid rescue experiment

We therefore conducted a second full factorial rescue-experiment using i.p. injections of physiological saline or 5 ng/g bw MC injection in mature females immediately followed by waterborne exposures of EtoH vehicle or 60 nM 17,20βP; Sigma-Aldrich, Oakville, ON, Canada) in EtoH vehicle (13) (final EtoH concentration in tank<0.001%) to address this possibility. Sample size in treatment groups ranged from n=18-27. Otherwise, the same experimental parameters described above for the PGF_2α_ rescue experiment were used.

### 2.4 Female zebrafish whole body steroid extractions and hormone quantification

Collected flash-frozen female zebrafish samples, which had been stored at –80 °C until processing, were used for steroid extraction and hormone analyses. Frozen samples were immediately transferred to a dry ice-filled container and individually ground into a fine powder using a pre-chilled mortar and pestle. Approximately 100 mg of powdered tissue was weighed into 1.5 mL microcentrifuge tubes and homogenized in 500 μL of ice-cold 50 mM Tris+PI using a Sonic Dismembrator Model 100 (Fisher Scientific, Ottawa, ON, Canada). A 400 μL aliquot of each homogenate was transferred to 16 ×150 mm borosilicate glass tubes and extracted with 2 mL (5 volumes of homogenate) of diethyl ether via vigorous vortexing for 45 s (3 x 15s, with 20s break in between). The mixture was left to stand for 15 min at room temperature to allow phase separation. Tubes were then placed in an ethanol/dry ice bath for ~30 s to freeze the aqueous phase, and the upper organic layer was carefully transferred to fresh, labeled glass vials. This extraction was repeated twice more with fresh diethyl ether, and all organic layers were pooled. Combined ether extracts were evaporated to dryness under a gentle nitrogen gas stream (2 PSI) using a PIERCE Reacti-Vap_TM_ lll, 18785 (Thermo Scientific Pierce Biotechnology, Waltham, MA, USA) at room temperature. Dried residues were reconstituted in 400 μL of the appropriate ELISA assay buffer (Cayman Chemical, Ann Arbor, MI, USA) and stored at –80 °C until analysis. Extraction efficiency was assessed by spiking homogenized samples with known quantities of ELISA standards for 17β-estradiol (E_2_; #501893, Cayman Chemical), Progesterone (P_4_; #482604, Cayman Chemical), and PGF_2α_ (#416014, Cayman Chemical), and recovery was calculated. Average recoveries for E_2_, P_4_, and PGF_2α_ were 81.2%, 104% and 92.2%. Quantification of E_2_, P_4_, and PGF_2α_ was performed using the ELISA kits (#501893, #582601, #516011; Cayman Chemical) and Assay sensitivities were 6 pg/mL for E_2_, and 10 pg/mL for P_4_ and PGF_2α_ as previously described (3). Cortisol was quantified using a commercial Cortisol immunoassay Kit (DetectX^®^ Cortisol Immunoassay Kit, Arbor Assays, Ann Arbor, MI, USA; sensitivity of 27.6 pg/ml), as previously described (20). All samples were run in duplicate, and individual replicates not meeting a cut-off threshold of<20% intra-assay coefficient of variation (CV) were excluded from subsequent analysis. Analyte concentrations were corrected for dilution and normalized first to the initial tissue weight (100 mg) used for extraction and secondly scaled to whole fish body weight.

### 2.5 Courtship behaviour analysis

The occurrence of several courtship behaviours in breeding pairs was analysed manually by two observers blind to treatment conditions. The behaviours included time to ovulation after barrier removal, the cumulative number of ovulation events during analysed recording time, the number of quivering events (flank-to-flank contact and jagged swimming followed by female flexion away), the number and cumulative duration during analysed recording time of male-initiated chasing events, the number of male-initiated touching events and number of events where male fully circled around the females. Specific behaviours were based on published ethograms (21) and had been previously analysed in our laboratories (2, 11, 22). Representative screenshots of described behavioural sequences are provided in **Supplementary File S1**. Both observers were trained on a subset of videos after which concordance scores in observations were quantified (**Supplementary File S2**).

### 2.6 Statistical analyses

All data were analysed and visualized using GraphPad Prism V10 (Boston, MA, USA). All raw data were initially assessed by Shapiro-Wilk test and Bartlett’s test to verify that assumptions of normal distribution and homoscedasticity were met. In cases where these assumptions were not met, standard transformations were used to improve normality. For the MC dose-response experiments, one-way ANOVA (homoscedastic) or Welch’s ANOVA (heteroscedastic) were used, whereas non-parametric data were analysed using a Kruskal-Wallis test. In cases of significant differences in the omnibus tests, relevant *post-hoc* tests were used (Tukey’s test for significant one-way ANOVAs or Dunn’s multiple comparison test for Kruskal-Wallis tests). In all cases a cut-off value of *P* < 0.05 was used for statistical significance. For the second experiment investigating effects of MC and PGF_2α_ on indices of reproductive success in a full factorial design, data were analysed as described above, but either two-way ANOVAs (normally distributed data) or a Kruskal-Wallis test with Scheirer-Ray-Hare extension (23) were used to assess significance of factors or their interaction. Effect size metric for statistical tests were calculated as follows: for one-way ANOVAs, η^2^ = Sum of squares (SS) between/SS total; for Kruskal Wallis test, *η^2H^*= (H - k +1)/n-k, where H is the Kruskal-Wallis statistic, k is the number of groups, and n is the total number of observations; for two-way ANOVA/Scheirer-Ray Hare test using ranked data, η^2p^ = SS effect/SS effect + SS error. To assess indices of pair breeding courtship behaviours in a dimensionality reduction approach, courtship indices form pairs with saline-injected female controls and pairs with 5 ng/g MC-injected females were analysed in a principal component analysis (PCA) using CLustVis (24). Variance scaling was applied to rows and the Singular Value Decomposition with imputation method used. Ellipses surrounding treatment groups represent a 95% probability for a treatment group-specific female to fall into based on dimensionality-reduced courtship behaviour indices. To assess the strength of relationship between individual pair-breeding behaviours and female reproductive success (number of viable eggs produced by a pair), linear regression analysis was conducted using GraphPad Prism and *P*-values for slopes significantly different form 0 as well as R^2^ values were computed.

## 3 Results

### 3.1 Acute Avpr1 inhibition decreased spawning and quivering behaviour irrespective of dose

**Figure 2:**
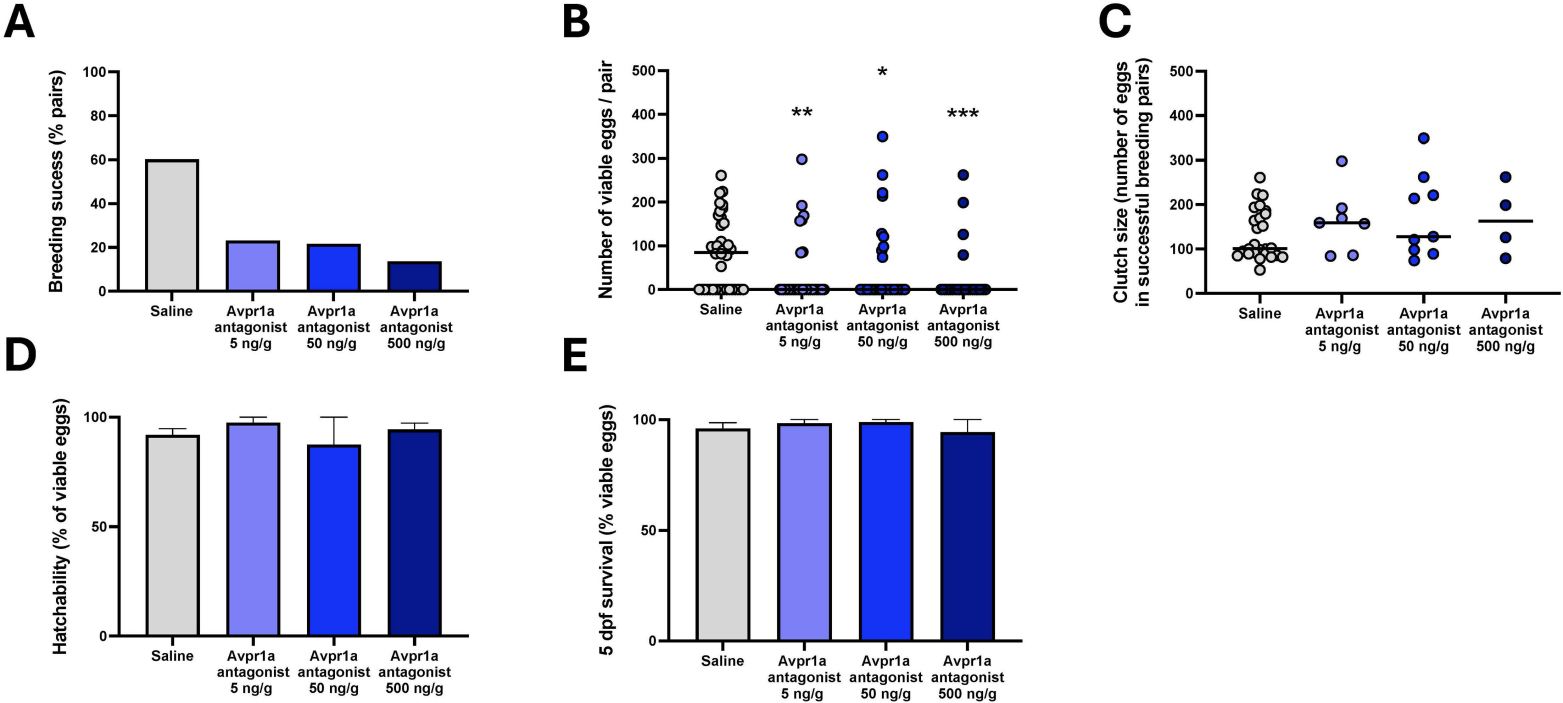
Indices of reproductive success in breeding pairs following i.p. injection of saline control, 5 ng/g bw, 50 ng/g bw and 500 ng/g bw MC in females. Breeding success in percent of all breeding pairs **(A)**, median number of viable eggs produced **(B)**, mean clutch size defined as mean number of eggs produced in successful breeding pairs **(C)**, median hatchability (± 95% CI) as percentage of viable eggs from replicate fertilized egg batches **(D)**, median 5 dpf survival (± 95% CI) as percentage of viable eggs from replicate experimental batches **(E)**. Individual data points are indicated in addition to means and medians for viable egg count data. Parametric data were analysed by one way-ANOVA, whereas non-parametric data were analysed by Kruskal-Wallis test. In cases of significant differences, *post-hoc* tests were used to resolve differences compared to saline control. Significant differences between saline control and treatment groups are indicated by *, ** and ***, indicating *P* < 0.05, *P* < 0.01, *P* < 0.001, respectively.

When using single acute injections of the MC several indices of female reproductive success were reduced in pair-breeding experiments (**Figure 2**). The percentage of successfully breeding zebrafish pairs was reduced from 60% in saline injected females to 20% in females injected with 5 ng/g and 50 ng/g MC and to 10% in females injected with 500 ng/g MC (**Figure 2A**). This reduction in successful breeding was evident in a significantly reduced number of viable eggs (**Figure 2B**; df=3, H = 17.72, *P* < 0.005, *η^2H^ =*0.06. Compared to saline-injected females, viable egg numbers were significantly reduced in females injected with 5 ng/g (P<0.01) 50 ng/g (P<0.05) and 500 ng/g (P<0.01) MC (**Figure 2B**). Conversely, injection of the specific OxtR antagonist L-368,899 using the same experimental design did not affect the number of viable eggs (df=2; H = 0.4972; P = 0.7799; **Supplementary File S3**).

**Figure 3:**
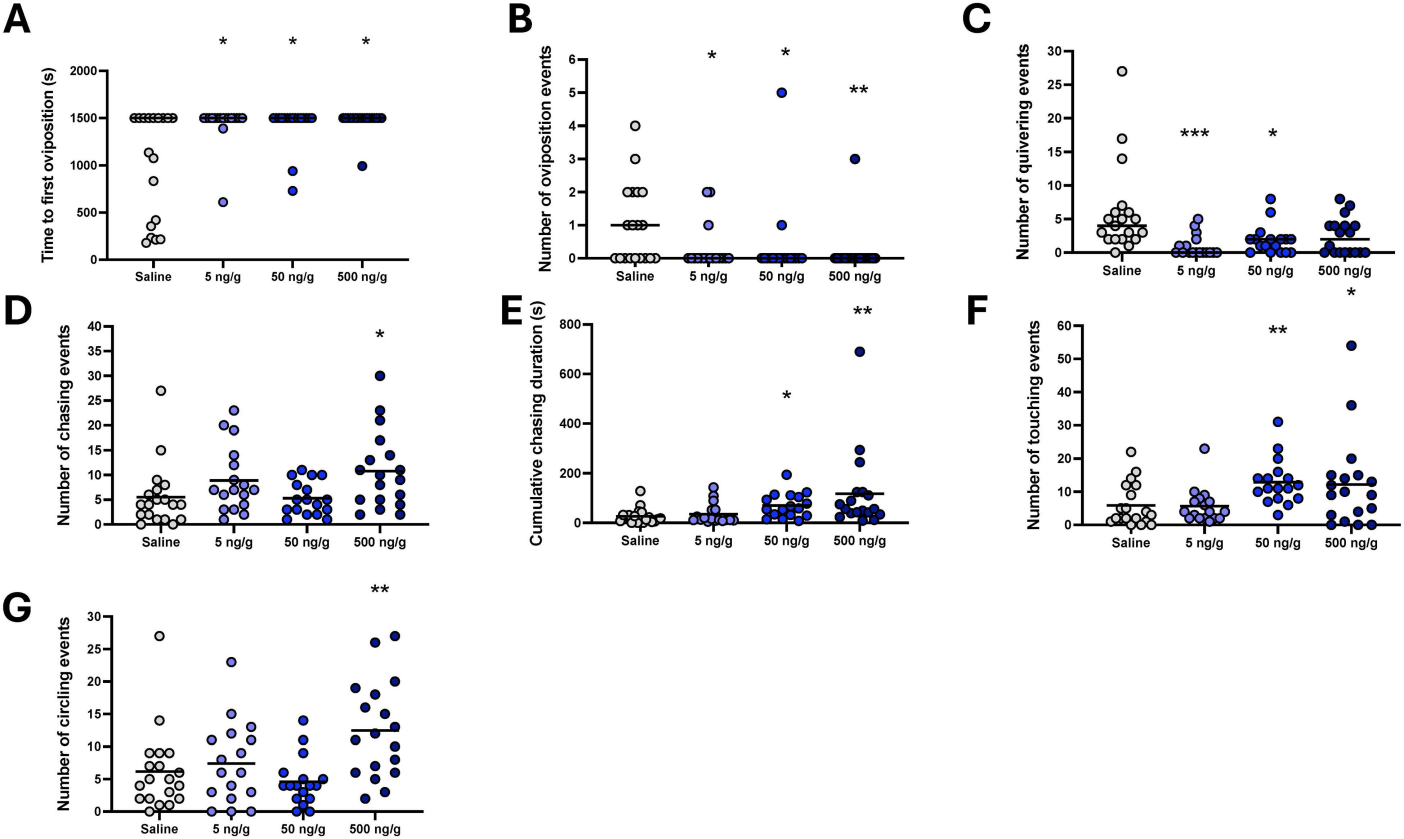
Indices of courtship behaviour in breeding pairs following i.p. injection of saline control, 5 ng/g bw, 50 ng/g bw and 500 ng/g bw MC in females. Median time to oviposition **(A)**, median number of oviposition events **(B)**, mean number of quivering events **(C)**, mean number of chasing events **(D)**, mean cumulative chasing time **(E)**, mean number of touching events **(F)** and mean number of circling events **(G)**. Individual data points are indicated in addition to means and medians. Parametric data were analysed by one way-ANOVA, whereas non-parametric data were analysed by Kruskal-Wallis test. In cases of significant differences, *post-hoc* tests were used to resolve differences compared to saline control. Significant differences between saline control and treatment groups are indicated by *, ** and ***, indicating *P* < 0.05, *P* < 0.01 and *P* < 0.001, respectively.

When considering clutch size in successful breeding pairs, no difference between treatment groups was found (**Figure 2C**; df=3, F = 0.87, *P* = 0.46). Neither hatching (**Figure 2D**; df=3, F = 0.8, *P* = 0.85) nor 5 dpf survival (**Figure 2E**; df=3, F = 4.19, *P* = 0.24) of eggs from breeding pairs was affected by treatment. When investigating indices of courtship behaviour and oviposition in recorded subsets of treatment groups (**Figure 3**), time to first oviposition (**Figure 3A**; df=3, H = 10.44, *P* < 0.001; *η^2H^ =* 0.08), number of oviposition events (**Figure 3B**; df=3, H = 13.02, *P* < 0.005; *η^2H^ =* 0.11) were significantly reduced compared to saline control by MC treatment irrespective of dose (*P* < 0.05). Quivering behaviour (**Figure 3C****;** df=3, H = 17.35, *P* < 0.001; *η^2H^ =* 0.18) was significantly reduced in pairs where females had been injected with 5 ng/g bw (P = 0.0001) and 50 ng/g (*P*<0.05) and marginally reduced compared to pairs with saline-injected control females (*P* = 0.06). The degree of correlation for scoring videos to analyse time to first oviposition (R^2^ = 0.88), number of oviposition events (R^2^ = 1) and quivering behaviour (R^2^ = 0.99) were high between observers who were blind to the treatment groups (**Supplementary File S2**).

### 3.2 Acute administration of MC increased courtship behaviour and whole-body progesterone at a higher dose

Increases in the number of chasing events (**Figure 3D**; df=3, F = 2.91, *P* = 0.04; η^2^ = 0.12), cumulative chasing duration (**Figure 3E**; df=3, F = 6.4, *P* < 0.001; η^2^ = 0.23), number of touching events (**Figure 3F**; df= F = 6.25, *P* < 0.001; η^2^ = 0.24) and the number of circling events (**Figure 3G**; df=3, F = 4.94, *P*0.039; η^2^ = 0.20) were observed in pairs where females had been injected with the highest concentrations of MC (500 ng/g bw) compared to pairs where females had been injected with saline (*P* < 0.05). For cumulative chasing duration (**Figure 3E**) and number of touching events (**Figure 3F**), pairs in which females had received the second highest concentration of MC (50 ng/g bw) also elicited significant increases compared to pairs with saline-injected females (*P* < 0.05). The inter-observer correlation scores on a subset of videos (**Supplementary File S2**) show high consistencies for scoring chasing events and circling events (R^2^ = 0.92), but comparatively lower for chasing time (R^2^ = 0.49) and touching events (R^2^ = 0.06).

**Figure 4:**
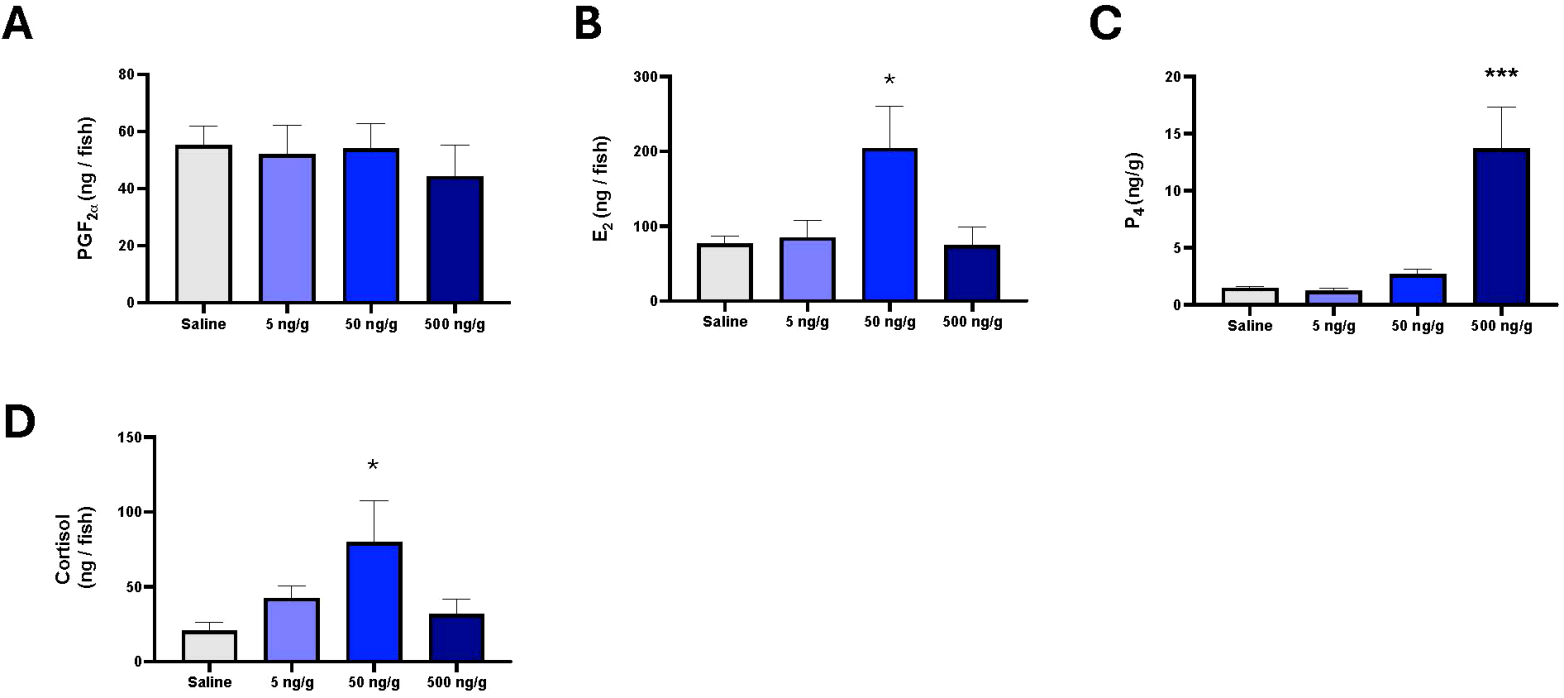
Mean whole body and body weight-normalized concentrations (± S.E.M.) of reproductive hormones estradiol **(A)**, prostaglandin F_2α_**(B)** and progesterone **(C)** and the stress hormone cortisol **(D)** in females i.p injected with saline, 5 ng/g bw, 50 ng/g bw or 500 ng/g bw MC. Data were analysed by one way-ANOVA. In cases of significant differences, post-hoc tests were used to resolve differences compared to saline control. Significant differences between saline control and treatment groups are indicated by * and ***, indicating *P* < 0.05 and *P* < 0.001, respectively.

Acute injection of MC in females did not alter whole body concentrations of reproductive hormones PGF_2α_ (**Figure 4A**; df=3, F = 0.18, *P* = 0.91). Conversely, whole body concentrations of E_2_ (**Figure 4B**; df=3, F = 4.18, *P* = 0.02, η^2^ = 0.024) and P_4_ (**Figure 4C**; df=3, F = 20.50, *P* < 0.0001; η^2^ = 0.75) increased significantly in 50 ng/g MC (**Figure 4B**; *P* < 0.05) and 500 ng/g MC (**Figure 4C**; *P* < 0.0001) groups when compared to saline injected female controls. Acute administration of MC significantly affected whole body cortisol concentrations (**Figure 4D**; df=3, F = 3.21, *P* < 0.04; η^2^ = 0.29), with a significant increase in cortisol in 50 ng/g MC injected females compared to saline controls (*P* < 0.05).

### 3.4 Acute co-injection of PGF_2α_ rescued Avpr1 MC-dependent reduction in oviposition

Given that we previously observed a decrease in ovarian PGF_2α_ and prostaglandin synthesis pathway gene expression that coincided with a reduction in viable egg number in female *avp*^-/-^ zebrafish compared to wildtypes (3), we tested the hypothesis that the observed reduction in female zebrafish injected with the MC is mediated by downstream action on PGF_2α_. We predicted that if PGF_2α_ signalling was a crucial downstream component of Avpr1 inhibition mediated effects on ovulation and/or courtship behaviour, then co-administration of PGF_2α_ would rescue the MC effect.

**Figure 5:**
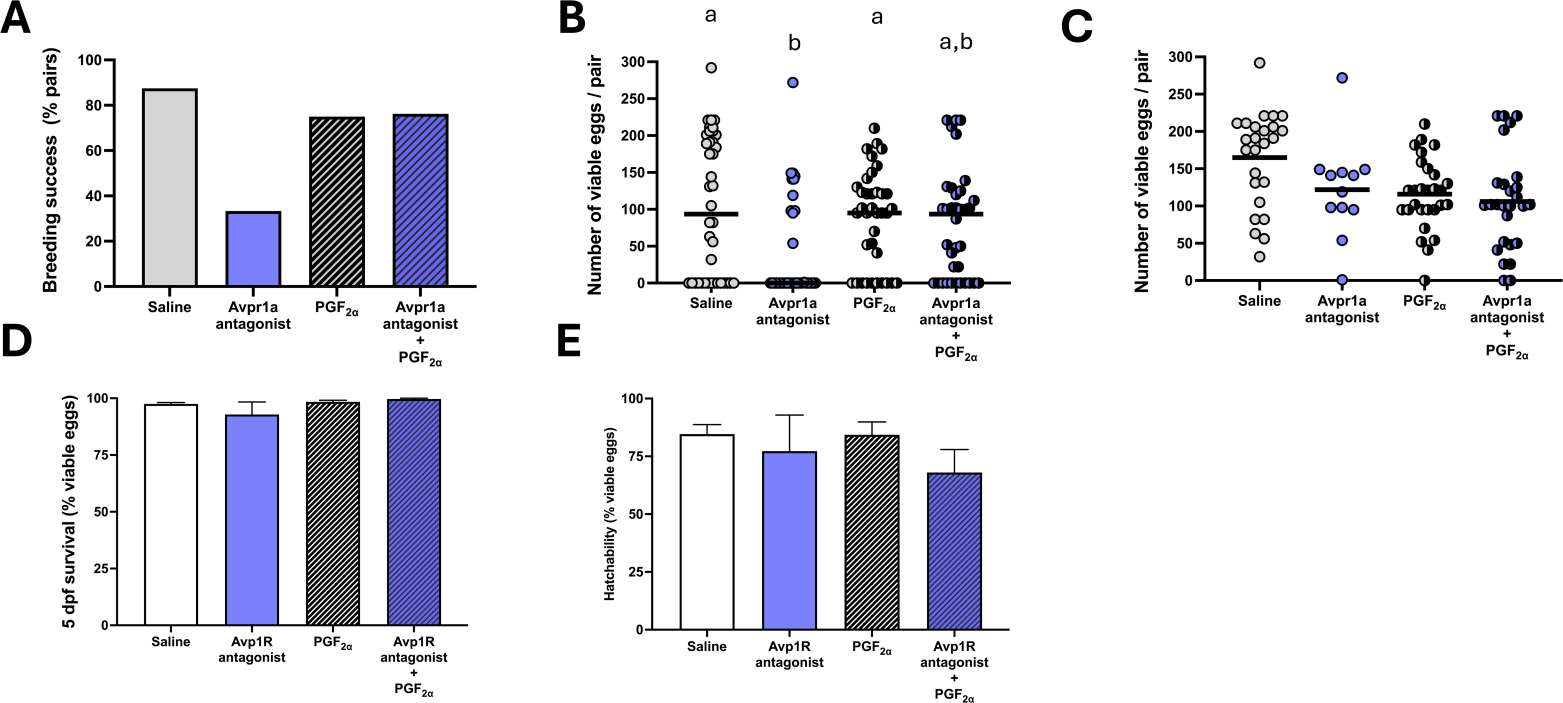
Indices of reproductive success in breeding pairs following i.p. injection of saline, 5 ng/g MC, 5 μg/g Prostaglandin F2α, and combined injection of 5 ng/g bw MC and 5 μg/g Prostaglandin F_2α_, in females. Breeding success in percent of all breeding pairs **(A)**, median number of viable eggs produced **(B)**, mean clutch size defined as mean number of eggs produced in successful breeding pairs **(C)**, median hatchability as percentage of viable eggs from replicate fertilized egg batches **(D)**, median 5 dpf survival as percentage of viable eggs from replicate experimental batches **(E)**. Individual data points are indicated in addition to means and medians for viable egg count data. Parametric data were analysed by two way-ANOVA, whereas non-parametric data were analysed by two-way ANOVA on ranks with Scheirer-Ray-Hare extension. In cases of significant differences, *post-hoc* tests were used to resolve differences compared to saline control. Significant differences between treatment groups (*P* < 0.05) are indicated by different letters.

In a full-factorial design, PGF_2α_ rescued the MC-dependent reduction in pair breeding success (**Figure 5A**). Saline-injected females successfully mated in >80% of assays, while application of 5 ng/g bw of MC reduced reproductive success to 33%. Females injected with 5 μg/g bw of PGF_2α_ mated in 75% of cases, as did females co-injected with 5 ng/g bw MC and PGF_2α._ A significant interaction effect of MC and PGF_2α_ treatment was found in the number of viable eggs produced (**Figure 5B**; df=1, H = 5.61, *P* = 0.02; η^2p^ = 0.08). *Post-hoc* analysis revealed that female injection of MC, but not PGF_2α_ treatment significantly reduced viable egg number produced in breeding pairs compared to pairs with saline injected control females (*P* < 0.05). Co-injection of MC and PGF_2α_ in females of breeding pairs resulted in viable egg numbers not significantly different from pairs with saline-injected control females. Clutch size (the number of viable eggs in successful breeding pairs, **Figure 5C**) was not affected by MC (df=1, F = 1.63, *P* = 0.21), but PGF_2α_ treatment, which significantly reduced clutch size (df=1, F = 5.3, *P* = 0.024; η^2p^ = 0.05). No significant interaction between MC treatment and PGF_2α_ treatment for was found for clutch size (df=1, F = 0.26, *P* = 0.61). Hatching percentage (**Figure 5D**) was not affected by MC treatment (df=1, H = 1.15, *P* = 0.28), PGF_2α_ treatment (df=1, H = 0.041, *P* = 0.83), or the interaction of MC and PGF_2α_ treatment (df=1, H = 0.63, *P* = 0.42). Similarly, survival to 5 dpf (**Figure 5E**) was not affected by MC treatment (df=1, H = 0.03, *P* = 0.86), PGF_2α_ treatment (df=1, H = 0.17, *P* = 0.67), or the interaction of MC and PGF_2α_ treatment (df=1, H = 0.10, *P* = 0.75).

Conversely, while waterborne exposure to 17,20βP appeared to normalize MC-dependent reduction in breeding success (**Supplementary File S4A**), no effect of MC, 17,20βP, or their interaction on viable egg number in breeding pairs was observed (**Supplementary File S4B**). 17,20βP exposure, but not MC treatment, or their interaction, significantly affected clutch size (df=1, F = 4.322, *P* = 0.042; η^2p^ = 0.08; **Supplementary File S4C**) and hatchability (df=1, F = 7.038, *P* = 0.011; η^2p^ = 0.12; **Supplementary File S4D**). 17,20βP exposure reduced clutch size and increased hatchability, respectively. Neither 17,20βP exposure, nor MC treatment, or their interaction, affected larval survival at 5 dpf (**Supplementary File S4E**).

### 3.5 Acute co-injection of PGF_2α_ does not rescue MC-dependent courtship behaviour

**Figure 6:**
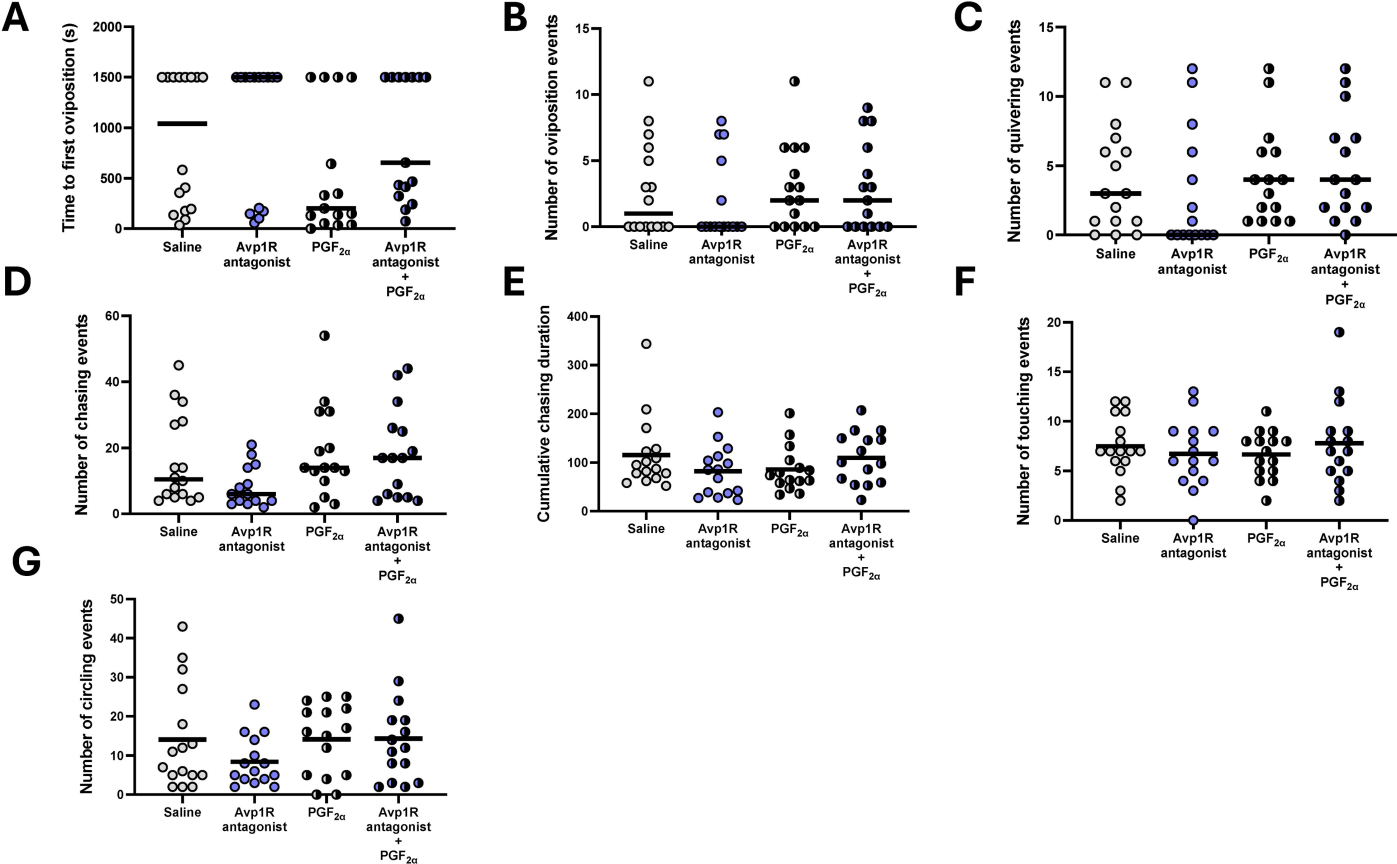
IIndices of reproductive success in breeding pairs following i.p. injection of saline, 5 ng/g MC, 5 mg/g Prostaglandin F_2α_, and combined injection of 5ng/g bw MC and 5 mg/g Prostaglandin F_2α_, in females. Median time to oviposition **(A)**, median number of oviposition events **(B)**, median number of quivering events **(C)**, median number of chasing events **(D)**, mean cumulative chasing time **(E)**, mean number of touching events **(F)** and median number of circling events **(G)**. Individual data points are indicated in addition to means and medians. Parametric data were analysed by two way-ANOVA, whereas non-parametric data were analysed by two-way ANOVA on ranks with Scheirer-Ray-Hare extension. *Post-hoc* tests were used to resolve differences in main factors or their interaction as described in the text.

Time to ovulation (**Figure 6A**) was marginally, but not significantly, increased by treatment with MC (df=1, H = 3.21, *P* = 0.07), and not affected by PGF_2α_ treatment (df=1, H = 1.72, *P* = 0.19) or their interaction (df=1, H = 0.97, *P* = 0.32). The number of oviposition events (**Figure 6B**) was not dependent on MC (df=1, H = 0.39, *P* = 0.53), PGF_2α_ (df=1, H = 1.24, *P* = 0.27) or their interaction (df=1, H = 0.29, *P* = 0.59). The number of quivering events (**Figure 6C**) was not affected by MC treatment (df=1, H = 0.62, *P* = 0.43), marginally, but not significantly increased by PGF_2α_ (df=1, H = 3.05, *P* = 0.08), and not dependent on their interaction (df=1, H = 1.14, *P* = 0.28). The number of chasing events (**Figure 6D**) was not significantly affected by the MC treatment (df=1, H = 0.32, *P* = 0.86), PGF_2α_ treatment (df=1, H = 2.57, *P* = 0.11) or their interaction (df=1, H = 0.90, *P* = 0.34). The cumulative chasing duration (**Figure 6E**) while not significantly affected by MC (df=1, F = 0.37, *P* = 0.54) or PGF_2α_ (df=1, F = 0.06, *P* = 0.544) treatments alone, significantly affected by their interaction (df=1, F = 4.69, *P* = 0.035; η^2p^ = 0.08). However, *post-hoc* analysis was unable to resolve specific differences between groups. The number of touching events (**Figure 6F**) was neither dependent on treatment with MC (df=1, F = 0.46, *P* = 0.83), PGF_2α_ (df=1, F = 0.19, *P* = 0.89) or their interaction (df=1, F = 1.23, *P* = 0.27). Similarly, the number of circling events (**Figure 6G**) was not affected by MC treatment (df=1, H = 0.88, *P* = 0.35), PGF_2α_ (df=1, H = 1.72, *P* = 0.19) or their interaction (df=1, H = 0.32, *P* = 0.57).

### 3.6 Dimensionality-reduced analysis of courtship behaviour fails to distinguish saline-injected from 5 ng/g MC-injected females

To investigate whether multiple assessed indices of pair-breeding courtship behaviour allow the separation of saline-injected control females and 5 ng/g MC-injected females, we investigated indices of courtship behaviours of both groups across experiments in a Principal Component Analysis (**Supplementary Files S5**, **S6**). PC1 and PC2 axes accounted for 53.5% and 13.6% of the variance respectively but did not distinguish treatment groups.

### 3.7 Courtship behaviours significantly but differentially correlate with viable egg numbers across experiments

**Figure 7:**
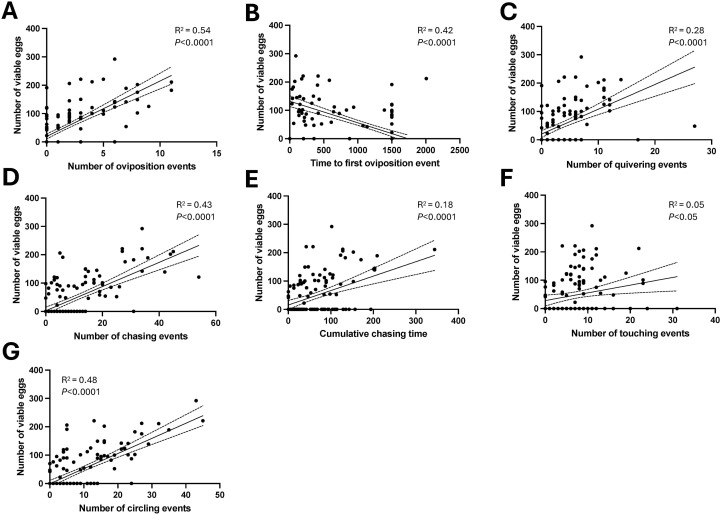
Linear regression analysis to assess whether individual courtship indices predict viable egg production by breeding pairs. Individual data from breeding pairs with saline-injected control females and 5 ng/g MC injected females are shown. Linear regression (solid lines) and 95% confidence interval ranges (dotted lines) are shown for number of viable eggs and number of oviposition events **(A)**, time to first oviposition event **(B)**, number of quivering events **(C)**, number of chasing events **(D)**, cumulative chasing time **(E)**, number of touching events **(F)**, number of circling events **(G)**.

To determine whether individual indices of pair-courtship behaviours differentially predict spawning success, we investigated the relationship between number of viable eggs spawned and courtship behaviour by linear regression analyses (**Figure 7**). Significant non-zero slopes were identified for all assessed courtship behaviours analysed form videos. However, the proportion of variance in the dependent variable (numbers of viable eggs) explained by the independent variable (specific courtship behaviour) revealed differences. The number of oviposition events (**Figure 7A**) correlated positively (R^2^ = 0.54), while the time to first oviposition event (**Figure 7B**) correlated negatively (R^2^ = 0.42) with the number of viable eggs produced in breeding pairs across all conditions tested. Positive correlations with the number of viable eggs of varying R^2^ were observed for the number of quivering events (**Figure 7C**; R^2^ = 0.28), number of chasing events (**Figure 7D**; R^2^ = 0.43) cumulative chasing time (**Figure 7E**; R^2^ = 0.18), number of touching events (**Figure 7F** R^2^ = 0.05) and number of circling events (**Figure 7D**; R^2^ = 0.48).

## 4 Discussion

### 4.1 Acute injection of MC in wild type female zebrafish recapitulates key aspects of reduced reproductive success in female avp ^-/-^ zebrafish

We here demonstrate that female Avp signalling is acutely involved in pair-breeding success in zebrafish, assessed by the number of spawned and fertilized, viable eggs. Because MC has been shown to effectively antagonize AvpR1 receptor in teleosts (10), and acute application of an OxtR-selective antagonist validated in zebrafish (18) did not affect the number of spawned and fertilized viable eggs, Avp effects on female zebrafish reproductive success are likely mediated via Avpr1 receptor signalling.

Our current acute pharmacological study using the Avpr1 receptor inhibitor MC confirms previous findings of a female-specific reproductive phenotype in zebrafish in *avp*^-/-^ knock-outs (4) which exhibited similar characteristics observed in our current study. In breeding pairs containing either *avp*^-/-^ and females acutely injected with MC at a dose as low as 5 ng/g, reductions in viable eggs were observed without affecting clutch size. Furthermore, in our current pharmacological study as well as our previous knock-out study, we observed a significant reduction in quivering behaviour which is associated with oviposition (21). While not assessed in our previous study, additionally analysed parameters related to oviposition (number of oviposition events, time to first oviposition) were affected at 5 ng/g and all other doses of MC tested. The acute nature of our pharmacological inhibition experiment confirms that the reported Avp action on female reproductive success is not specific to genetic ablation, which may suffer from confounds of developmental alterations in absence of a functional Avp system and observed life-long hypercortisolism (3). Indeed, while acute MC injection affected whole body cortisol in females, a significant increase compared to saline control was only observed at 50 ng/g bw injection, but not 5 ng/g or 500 ng/g MC injections. While we did not assess whole body cortisol concentrations in no-injection control fish, saline injected fish pair-breeding success rates (between 60-80 percent) and viable egg production (median of ~100 viable eggs) in our experiments in line with maximal rates observed in the facility for non-injected WT fish mating for colony maintenance, suggesting injection stress did not diminish this measure of female reproductive success. The current pharmacological manipulations also temporally place the role of Avp to previously suspected effects on ovulation and spawning: because our injection protocol was conducted at onset of lights, the reproductive effects of female Avp system can be placed after the Luteinizing hormone (LH) surge reported to occur several hours prior (25) and in the periovulatory and spawning time. This is further supported by the consistent significant reduction in quivering behaviour observed in genetic ablation of the Avp system (3) and the current acute pharmacological inhibition of the Avpr1 receptors. The current study also revealed a significant reduction in the number of oviposition events and time to first oviposition, all at the lowest concentration of 5 ng/g MC shown to significantly reduce viable egg release in breeding pairs. While central AvpR1-depednent neuromodulation and/or pituitary involvement cannot be excluded in systemic acute application of MC (4), it is noteworthy that collectively courtship behaviours did not distinguish 5 ng/g MC injected and saline injected females, and that pioneering evidence for central roles for Avp in female spawning in some, but not all fishes is controversial due to the fact that only high doses of central Avp administration which match peripheral doses have been shown to elicit female spawning, an effect thus ascribed to a likely peripheral site of action (26).

### 4.2 Avp likely modulates female zebrafish reproductive success via Avpr1, possibly at the level of the ovary

MC has been developed as specific mammalian Avpr1a antagonist (27). While its specificity among nonapeptide receptors in zebrafish or fish in general has, in contrast to the oxytocin receptor antagonist L-368,899 (18), not been formally validated by heterologous receptor expression assays, it has been shown to antagonize AvpR1-type receptors in white sucker (10). MC has also been widely used in comparative work in fishes, in some cases to delineate nonapeptide receptor contributions (2, 11, 13–15).Thus, our study demonstrates that the previously observed female-specific consequences on zebrafish reproductive fitness observed in our *avp*^-/-^ model are likely principally mediated by Avpr1 type receptors, although a contributing role for other nonapeptide receptors cannot be excluded in absence of teleost nonapeptide receptor specificity data for MC. Since acute female injection of the selective OxtR receptor antagonist L-368,899 at similar doses to MC did not result in a reduction of viable eggs in breeding pairs, it is unlikely that the effect of MC is partially mediated by Oxtr. This important, as some evidence for Avp-dependent stimulation of OxtR receptors has been linked to ovarian synthesis of PGF_2α_ in oviparous guppies (28). Current efforts in our lab are on the way to generate different knockouts for (paralogous) nonapeptide receptors in zebrafish. These mutants will allow to provide additional support for AvpR1 action in MC-dependent suppression of metrics of female reproductive success, and possibly resolve Avpr1aa and Avpr1ab paralogue specific contributions.

As discussed, the Avp system may affect the HPG axis in fishes via central neuromodulation, pituitary hormone release, and/or (paracrine) ovarian action (4). Ovarian *avp* expression has been described to increase in post-vitellogenic follicles in rainbow trout (*Oncorhynchus mykiss*), suggesting a critical role in post-vitellogenic ovarian function (29). Studies in seasonally breeding Asian stinging catfish females, which exhibit synchronous ovarian oogenesis, revealed increased ovarian Avp peptide levels in plasma and ovaries during the spawning phase compared to preparatory and pre-spawning phases, in line with placing the acute action of Avp in the periovulatory and/or spawning period (30). Interestingly, *avpr1a* receptor expression has recently been localized to follicular cells in Asian stinging catfish ovaries (31) and seabass (*D. labrax*) (32) While evidence for the localization of the Avpr1 paralogue transcript *avpr1aa* in zebrafish thecal cells exists within a single cell sequencing dataset for zebrafish ovaries (33)) preliminary work in our lab confirmed ovarian expression of *avpr1aa* and *avpr1ab* paralogues by *real-time* RT-PCR analysis at the whole tissue level (unpublished results). Thus, molecular evidence suggests that MC could directly act at the ovarian level to modulate periovulatory oocyte development and spawning in zebrafish. However, given that *avpr1*-type transcripts in fish species including zebrafish have been identified centrally and, in the pituitary (3), the contribution of central and/or pituitary level modes of actions via AvpR1-type receptors cannot be formally excluded using a pharmacological *in vivo* approach. Future studies using Avp/MC treatment of cultured zebrafish ovarian tissue culture are necessary to further assign tissue-specific contributions.

### 4.3 MC-dependent modulation of female reproductive success in zebrafish may be linked to downstream action ofPGF_2α_

Because we had previously observed significant reductions in ovarian PGF_2α_ concentration and key genes implicated in the PGF_2α_ synthesis pathway from arachidonic acid (3) in female *avp ^-/-^* zebrafish, we measured whole body PGF_2α_ as well as other female reproductive hormones involved in oogenesis, ovulation and spawning. In contrast to previous observations in ovaries of *avp*^-/-^ mutants (3), we did not observe significant changes in PGF_2α_. Given that other tissues have been reported sites of PGF_2α_ synthesis in fishes (34–36), it is possible that whole body resolution failed to capture more localized, tissue-specific effects at the ovarian (3) or circulating level. In female zebrafish ovaries, PGF_2α_ dynamics have been investigated in detail *in vivo* and *in vitro* (37, 38). While some studies revealed a significant increase in spontaneously spawning compared to non-spawning female zebrafish at 8 am (lights on) compared to pre-ovulatory levels at 12:00am (37), a more detailed time course analysis of the ovulatory period did not identify changes in ovarian PGF_2α_, suggesting a possibly extremely transient nature of PGF_2α_ induction at spawning (38). Gene expression analysis of key transcripts involved in AA mobilization and prostaglandin biosynthesis found to be down-regulated in *avp*^-/-^ females (3), were shown to exhibit tight developmental and temporary control, exemplified by a higher expression of *ptgs2* in mature compared to earlier stage follicles, and acute increases in the periovulatory period just prior to lights on compared to previous timepoints (37, 38). Recent manipulative *in vitro* studies in Asian catfish ovaries provide direct evidence for a role of Avp in PGF_2α_ synthesis (39). In pre-spawning ovaries incubated *in vitro*, Avp significantly induced PGF_2α_ tissue concentrations at all Avp concentrations tested (1-1000 nM), an effect significantly reduced by Avpr1, but not Avpr2 receptor antagonist co-incubation (39).

Therefore, to determine whether the observed acute pharmacological effects of Avpr1 blockage observed in our study were dependent on downstream effects on PGF_2α_ in mating zebrafish, we co-injected the lowest tested effective dose of the MC(5 ng/g bw) with 5 μg/g PGF_2α_, previously reported to induce ovulation and courtship behaviours in goldfish, *Carassius auratus* (19). As predicted, co-injection of PGF_2α_ partially rescued the observed reproductive phenotype, increasing the amount of viable spawned eggs to levels not different from control or PGF_2α_ injection alone without affecting clutch size. The rescue effect of PGF_2α_ may occur at either the ovulation or oviposition/spawning level, or both. In line with a direct role for prostaglandins in ovulation in zebrafish, treatment of fully grown follicles with indomethacin, a non- selective cyclooxygenase inhibitor, inhibited oocyte maturation assessed by germinal vesicle breakdown (37). While a mechanistic role for PGF_2α_ in inflammation-like responses involving matrix metalloproteinases in follicle rupture and ovulation processes is well-described in mammals, comparative research in zebrafish and other fishes is beginning to explore the conservation of this mechanism (40–43). While a role for nonapeptides and PGF_2α_ in mammalian uterine contractions are well-established, comparative roles in teleost fish ovary contraction linked to ovulation (44) are currently unknown and warrant future study. While the dose for i.p. injection of PGF_2α_ injection was selected based on a study investigating the effect of acute PGF_2α_ administration on female goldfish ovulation (19), the transient nature and interindividual variability of PGF_2α_ increase in the zebrafish spawning period (37, 38, 40) make it difficult to assess endogenous ovarian and or circulating concentrations and relate them to internal concentrations obtained through the i.p. injection. As such, future studies exploring endogenous ovarian and plasma PGF_2α_ dynamics in female zebrafish, as well as dose-response studies for i.p. PGF_2α_ administration and their link to ovarian circulating PGF_2α_ concentrations are desirable. Furthermore, *in vitro* approaches similar to those demonstrating a role of Avp and AvpR1 antagonists on ovarian PGF_2α_ synthesis in catfish (39) are needed in zebrafish.

### 4.4 Avp effects on PGF_2α_ may involve other upstream systems involved in oocyte maturation

Work in the Asian sting catfish model has demonstrated that in addition to PGF_2α_, the maturation-inducing steroid 17α,20β-dihydroxy-4-pregnen-3-one (17,20P), which is stimulated by the LH surge and critical in the resumption of meiosis and final maturation of oocytes including germinal vesical breakdown (GVBD) in oocytes, is also stimulated by Avp *in vitro* (6). In zebrafish, 17,20P itself induces ovulation in both solitary females and mixed sex pairs, but only in the latter does it also lead to ovulation, likely due to the requirement of male-specific courtship signals (40). In females treated with 17,20P and housed with a male, ovarian PGF_2α_ concentration is increased immediately after spawning (40), coincident with a transient significant increase in *ptgs2* expression 2h after 17,20P exposure (40). In light of this evidence, a recent review on the role of prostaglandins in the teleost ovulation concluded that ‘it is reasonable to assume that 17,20βP, which is synthesized in the granulosa cells of the preovulatory follicles in response to LH surge and activates Pgr, is a critical upstream mediator of PG synthesis’ (42). It is therefore conceivable, that Avpr1-dependent and PGF_2α_-mediated effects may involve further upstream action on 17,20P maturation inducing steroid as intermediary. In support of an additional upstream role of Avp in ovulation and acquisition of oocyte competency in zebrafish, a significant induction of *pgrmc1* and *pgrmc2*, components of membrane bound progesterone receptors, was identified in ovaries of *avp*^-/-^ knock-out zebrafish (3). These transcripts play a role in fine-tuning oocyte plasma membrane expression of membrane progesterone receptor (mPRa), which is critically involved mediating oocyte maturation via post LH surge action of 17,20P, the maturation inducing steroid in zebrafish (45). At least Pgrmc1 is also involved in maintaining oestrogen-dependent meiotic oocyte arrest prior to ovulation (46), suggesting that negative regulation by Avp may be involved in de-repression of meiotic arrest (3). However, our subsequent test of this hypothesis in form of an additional rescue-type experiment using MC and 17,20βP (**Supplementary File S4**) revealed that at least at the timepoint of MC application investigated (lights on), reduced spawning and is not dependent on upstream effects on 17,20βP.

### 4.5 The MC-dependent reduction in spawning behaviours linked to oviposition are not rescued by PGF_2α_

It is well established in goldfish that the periovulatory release of PGF_2α_ into female circulation coordinate female spawning behaviour with ovulation processes (47). Furthermore the excretion of peri-ovulatory circulating PGF_2α_ via urine has been demonstrated to act as releaser pheromone to also synchronise male courtship behaviour, in goldfish, but also zebrafish (48, 49). We therefore hypothesized that PGF_2α_ injection would secondarily rescue specific courtship behaviours linked to spawning. In breeding pairs containing both *avp*^-/-^ females (3) and MC-injected females (this study), we observed a consistent significant reduction in quivering behaviour, which is linked to oviposition and synchronized gamete release (21). In the rescue experiment, we observed that quivering behaviour, as well as parameters previously not measured in the *avp*^-/-^ but directly related to oviposition (number of oviposition events, time to first oviposition) showed a tendency towards increase compared to pairs with MC-injected females and normalization compared to pairs containing saline or PGF2_2α_-injected females. However, while these tendencies appear more specific to more immediate ovulation/spawning behaviours compared to other courtship indices analysed, the interaction term in the full-factorial analysis is, in contrast to the number of viable eggs produced by breeding pairs, not significant. Because courtship behaviour endpoints are more variable in nature compared to the quantification of spawned viable eggs, and because many of the behavioural data are not-normally distributed, it is possible that the Scheirer-Ray-Hare extension test, a non-parametric equivalent to two-way ANOVA analyses designed to test for interaction terms of two factors, lacked sufficient power to resolve the described differences in median. While improvements in video analyses allow for high quality scoring and interpretation of blind courtship behaviour analyses, the manual assessment inherently suffers from limitations in scope. Different sample sizes linked to available batch sizes and some differences in inter-rater concordance in scoring specific behaviours, especially touching behaviour, represent further limitations and warrant caution in the interpretation. Therefore, in the current absence of reliable automated courtship behaviour analyses in zebrafish, future studies with larger sample sizes are warranted to specifically assess the rescue of spawning specific behaviours.

When analysing the effect of MC on courtship behaviours, it is noteworthy that in the initial dose-response study of i.p. injection (Experiment 1), behaviour indices were affected in a dose-dependent manner. While an increase in time to first oviposition, a reduction in the number of oviposition events and the number of quivering behaviours were observed at 5 ng/g, the lowest dose tested to result in a reduction in pair breeding success and number of viable eggs produced, an increase in indices of chasing, circling and touching was observed exclusively at the higher doses of MC tested. These findings may argue for acute 5ng/g MC dose effects on behaviours more directly linked to oviposition and spawning. Indeed, among the endpoints of the video analyses obtained, the number of ovulation events was most strongly correlated with the number of viable eggs produced (R^2^; 0.54; **Figure 7**).

Conversely, global analysis of the overall effect of 5 ng/g MC on all female zebrafish courtship behaviour showed that these behavioural endpoints analysed were not sufficient to discern the 5 ng/g MC group from the saline-injected controls (**Supplementary Files S5**–**6**) in line with the suggestion that more acute spawning-specific behaviours, rather than overall courtship behaviours are under Avpr1 control. If verified in future experiments, these results would point to the fact that ovarian Avp actions liberating PGF_2α_ with subsequent transport to and action in the female brain, rather than direct central Avpr1 receptor dependent neuromodulation, are responsible for the specific, spawning specific behavioural effects such as quivering. The fact that higher concentration increased courtship indices in pairs that can be linked to male-initiated aspects of courtship behaviour (chasing, circling, touching) merits further investigation. These findings may indicate higher male efforts to engage in courtship behaviour, possibly a compensation to lower female responsiveness. Analysis of female circulating PGF_2α_ in 5 ng/g MC-injected and saline-injected females would be useful to address this possibility as ovulation-dependent rapid increases in circulating PGF_2α._ have been linked to synchronizing ovulation with female reproductive behaviours in another cyprinid species, the goldfish (48).

### 4.6 General conclusions and future directions in the functional investigation of Avp in zebrafish reproduction

In a wider context, the current work demonstrates the utility and feasibility of acute rescue-type experiments in zebrafish ovulation/spawning in pair mating conditions. This is important, as in zebrafish, *in vitro* work is hampered by the fact that in contrast to other fish species spontaneous ovulation does not occur *in vitro* and is subject to 17,20P stimulation (40). Furthermore, the set-up allows the specific testing of mechanistic hypotheses in an appropriate sociosexual context of courtship behaviour necessary for spawning following 17,20P induced ovulation (40). Using this approach, the current work answers directly to the call for more comparative investigation of Avp function in female reproduction in the diverse vertebrate class of teleost fishes (6), and points to an important role for Avp in female reproductive success in a model species with non-synchronous ovarian development. As such, our current *in vivo* work adds to previous mechanistic *in vitro* work in the Asian sting catfish and suggests an evolutionarily conserved role of Avp in female reproductive success among teleost fishes. However, future work is necessary to fully support this hypothesis. Given the hypothesized ovarian site of action, future work is needed to (co-)localize Avp, AvpR1a and enzymes involved in PGF_2α_ synthesis enzymes at the protein or gene expression level in the zebrafish ovary. Furthermore, especially given the transient nature and inter-individual differences of ovarian PGF_2α_ dynamics in female zebrafish (37, 38, 40), detailed time-course data would allow to strengthen the evidence for dynamic action of Avp on ovarian PGF_2α_ in zebrafish. Lastly, comparative *in vitro* study of ovarian Avp/MC action on PGF_2α_ synthesis and (PGF_2α-_dependent effects) on ovarian maturation and possibly ovarian contraction (44) are warranted in zebrafish and zebrafish nonapeptide receptor knock-outs.

In the evolutionary context of nonapeptide function, our work implicates Avp in female reproduction by affecting spawning via Avpr1-type receptors. The recent identification of *Avpr1ab* transcripts in follicular cell layers in the Asian sting catfish (31) and the seabass (32)), provides evidence for the Avpr1ab rather than the Avpr1aa paralogue as mediator of Avp effects on PGF_2α_. However, single a single cell data set of zebrafish ovaries only identified *avpr1aa* paralogue and localized its expression to thecal cells. It is noteworthy that recent studies zebrafish revealed that not only Avp, but also oxytocin (Oxt), bind to Avpr1ab with high affinity compared to the paralogous Avpr1aa (18). This suggests that both Avp and Oxt nonapeptides may be involved in regulating spawning via Avpr1a-type receptors, at least in some species. In absence of formal comparative investigation of MC antagonism of receptors other than AvpR1, in zebrafish, teleosts, and other non-mammalian species, future receptor knock-out studies targeting *avpr1aa, avpr1ab, oxtra, oxtrb* in zebrafish are necessary to fully delineate possible crosstalk between nonapeptide systems and assign receptor paralogue-specific contributions to female zebrafish reproduction. To this effect, female i.p. injection of MC and reproductive endpoints used in the current study should be applied in the knock-outs to unambiguously link specific receptors to Avp/MC effects. Nonapeptide receptor knock-outs will furthermore be useful in delineating receptors involved in Avp/MC regulation of PGF_2α_ synthesis in zebrafish ovaries *in vitro*.

In other teleost fishes, less potent effects of Oxt compared to Avp on female reproductive physiology at the level of the ovary have generally been reported. For example, compared to Avp, Oxt only weakly stimulated PGF_2α_ and 17.20P synthesis and GVBD in Asian sting catfish (6). In line with this suggestion and although not explicitly tested, no ovulation or spawning phenotypes have been reported in generated *oxt*^-/-^ zebrafish and medaka at the whole animal level (50, 51). Injecting a selective OxtR antagonist to females did, in contrast ot MC, not reduce pair spawning success, in our current study supporting a predominant role for Avp in zebrafish as well. Future comparative studies are warranted to assess the evolutionary conservation of these differential nonapeptide effects on female reproduction in fishes, as well as anamniotes such as frogs (16).

While our current study focused on the role of Avp in female zebrafish based on the finding that *avp*^-/-^ females, but not males, exhibit a phenotype indicative of reduced reproductive fitness (3), previous work has nevertheless demonstrated acute effects of Avp on male zebrafish courtship (2), spermatogenesis and steroidogenesis (52). Using an *in vivo* pharmacological rescue approach in line with the experiments described in this study, future studies could test whether MC-dependent reduction in male courtship behaviours (2) can be rescued by 11-KT injection, recently described to be stimulated by Avp in male testes explants and mediators of Avp’s effect on spermatogenesis (5).

## Data Availability

The raw data supporting the conclusions of this article will be made available by the authors, without undue reservation.

## Supplementary material

SUPPLEMENTAL FILE S1 Representative screenshots of specific courtship behavioural sequences captured and analyzed. **(A)** Spawning; **(B)** Quivering; **(D)** Chasing; **(D)** Touching; **(E)** Circling.

SUPPLEMENTAL FILE S2 Concordance rates in blind manual courtship behaviour analysis between both observers across experiments 1 and 2. Linear regression with 95% confidence intervals are plotted for **(A)** time to oviposition, **(B)** number of oviposition events, **(C)** number of quivering events, **(D)** number of chasing events, **(E)** cumulative chasing time, **(F)** number of touching events and **(G)** number of circling events.

SUPPLEMENTAL FILE S3 Index of reproductive success in breeding pairs following i.p. injection of saline control, 50 ng/g bw and 500 ng/g bw of the OxtR receptor antagonist L-368,899 in females. The median number of viable eggs produced is shown. Individual data points are indicated in addition medians for viable egg count data. Data was non-parametric data and analyzed by Kruskal-Wallis test.

SUPPLEMENTAL FILE S4 Indices of reproductive success in breeding pairs following i.p. injection of physiological saline or 5 ng/g MC and EtoH vehicle or 60 nM 17,20bP, Breeding success in percent of all breeding pairs **(A)**, median number of viable eggs produced **(B)**, mean clutch size defined as mean number of eggs produced in successful breeding pairs **(C)**, median hatchability as percentage of viable eggs from replicate fertilized egg batches **(D)**, median 5 dpf survival as percentage of viable eggs from replicate experimental batches **(E)**. Individual data points are indicated in addition to means and medians for viable egg count data. Parametric data were analyzed by two way-ANOVA, whereas non-parametric data were analyzed by two-way ANOVA on ranks with Scheirer-Ray-Hare extension. In cases of significant differences, post-hoc tests were used to resolve differences compared to saline control. Significant differences between treatment groups (*P*<0.05) are indicated by different letters.

SUPPLEMENTAL FILE S5 Principal Component Analysis of all assessed courtship behaviours of physiological saline i.p.-injected and 5 ng/g i.p.-injected MC groups across experiments 1 and 2. Unit variance scaling is applied to rows; SVD with imputation is used to calculate principal components. X and Y axis show principal component 1 and principal component 2 that explain 53.5% and 13.6% of the total variance, respectively. Prediction ellipses are such that with probability 0.95, a new observation from the same group will fall inside the ellipse. n=67 datapoints.

SUPPLEMENTAL FILE S5 Axis loading of Principal Component Analysis of all assessed courtship behaviours of physiological saline i.p. injected and 5 ng/g bw MC i.p. injected groups across experiments 1 and 2.
