# Supplementary figures and images for: Acute administration of Manning compound during the spawning period reduces reproductive success in female zebrafish
Source: Front Endocrinol (Lausanne). 2026 Apr 30;17:1771879. doi: 10.3389/fendo.2026.1771879 (PMC13171328; doi:10.3389/fendo.2026.1771879)

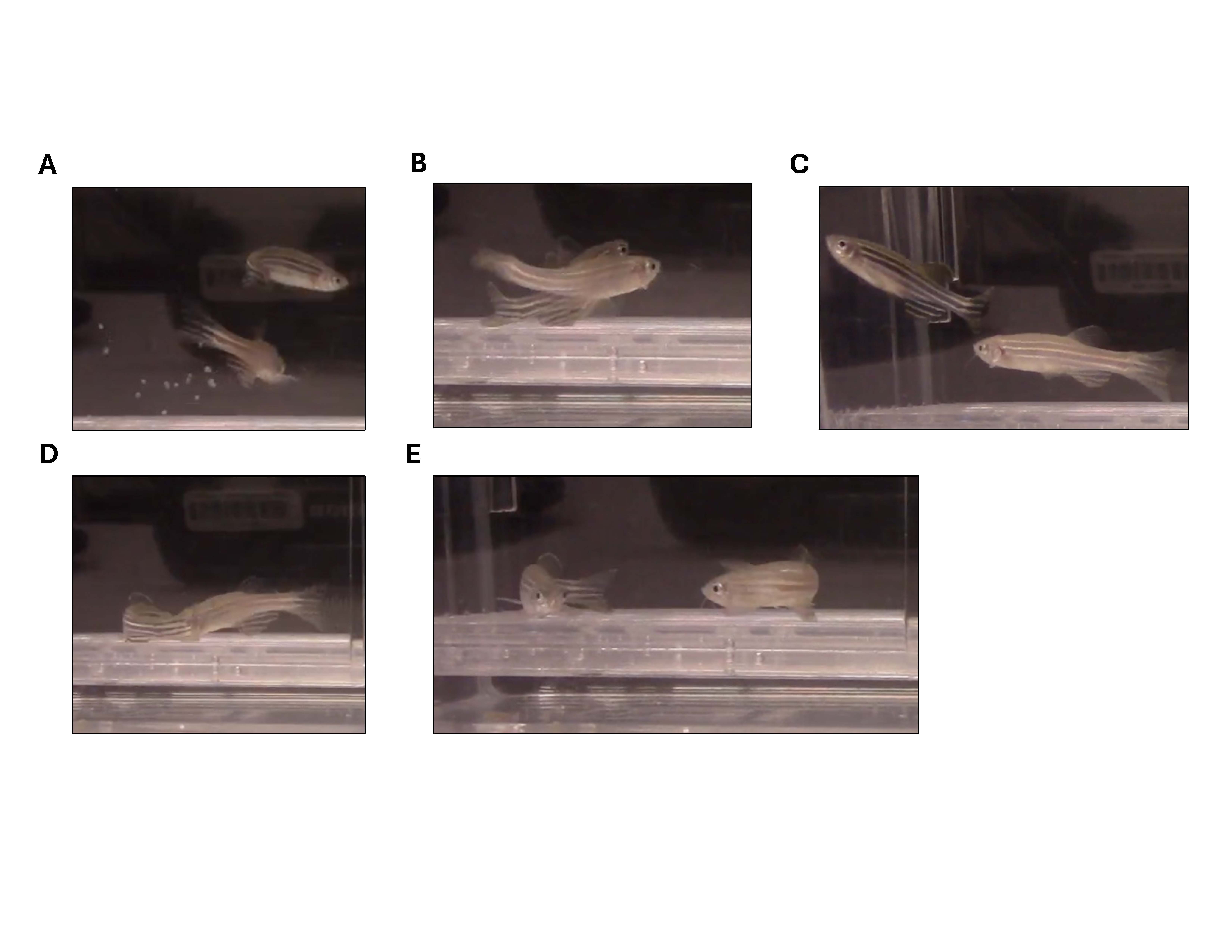

Supplement: SUPPLEMENTAL FILE S1 — Representative screenshots of specific courtship behavioural sequences captured and analyzed. (A) Spawning; (B) Quivering; (D) Chasing; (D) Touching; (E) Circling. [file Image1.jpeg]

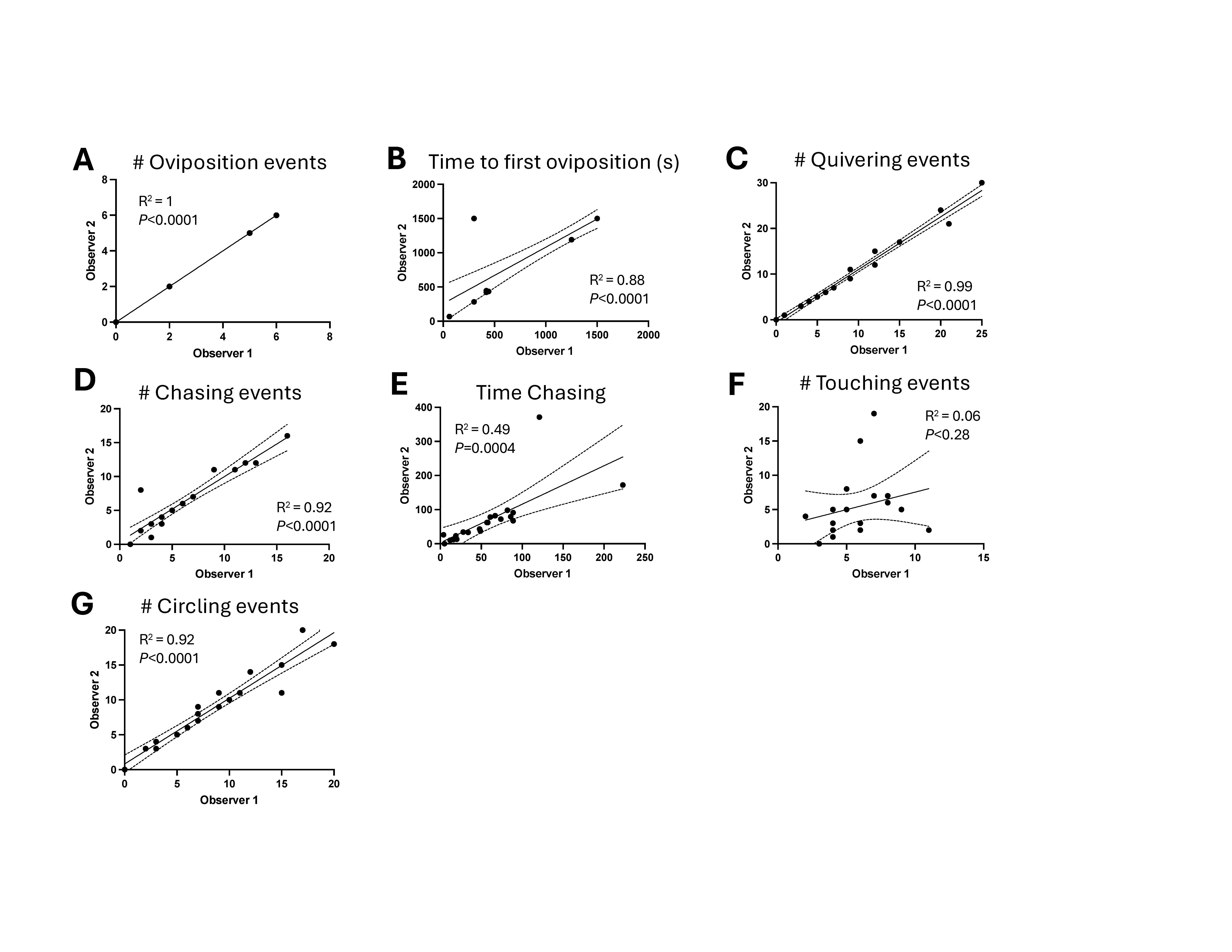

Supplement: SUPPLEMENTAL FILE S2 — Concordance rates in blind manual courtship behaviour analysis between both observers across experiments 1 and 2. Linear regression with 95% confidence intervals are plotted for (A) time to oviposition, (B) number of oviposition events, (C) number of quivering events, (D) number of chasing events, (E) cumulative chasing time, (F) number of touching events and (G) number of circling events. [file Image2.jpeg]

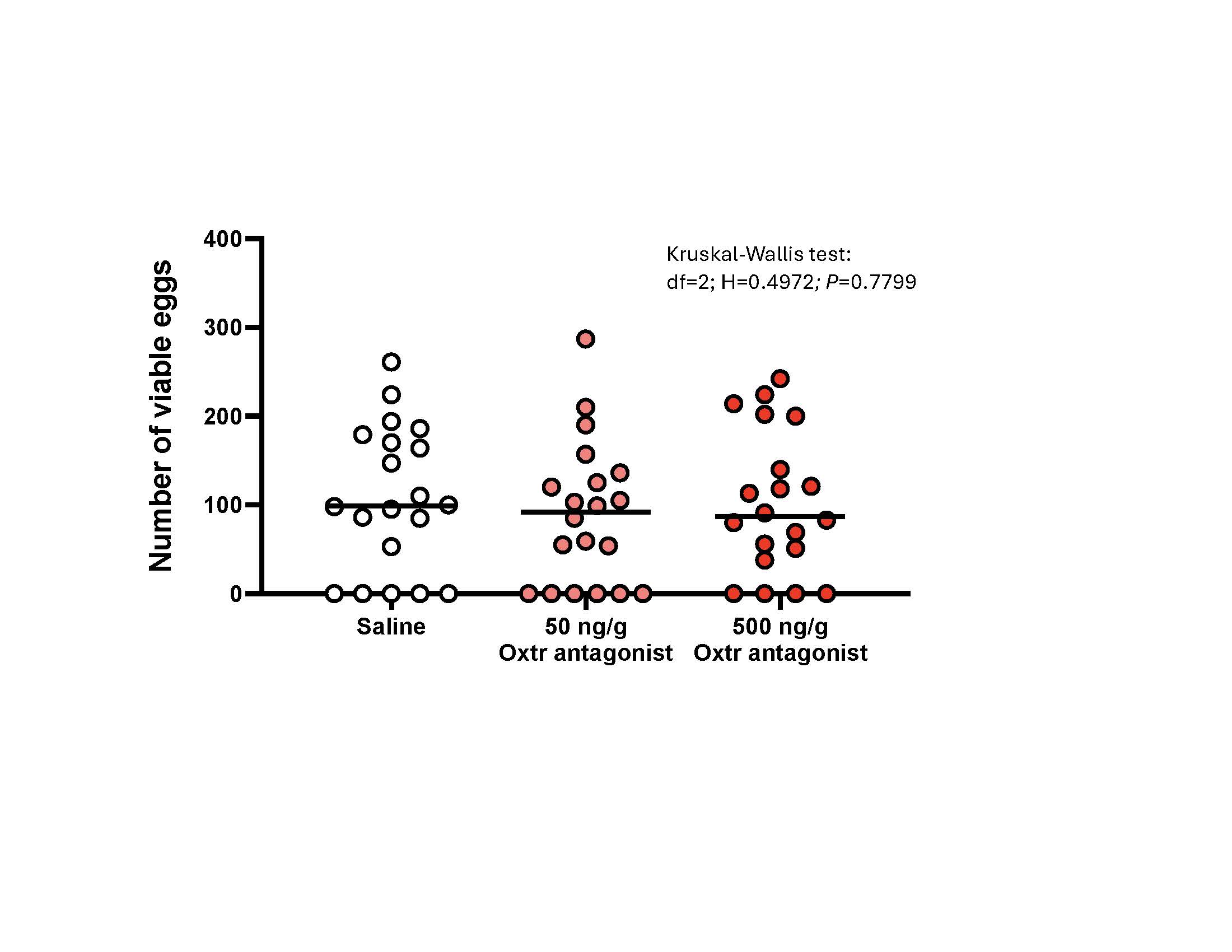

Supplement: SUPPLEMENTAL FILE S3 — Index of reproductive success in breeding pairs following i.p. injection of saline control, 50 ng/g bw and 500 ng/g bw of the OxtR receptor antagonist L-368,899 in females. The median number of viable eggs produced is shown. Individual data points are indicated in addition medians for viable egg count data. Data was non-parametric data and analyzed by Kruskal-Wallis test. [file Image3.jpeg]

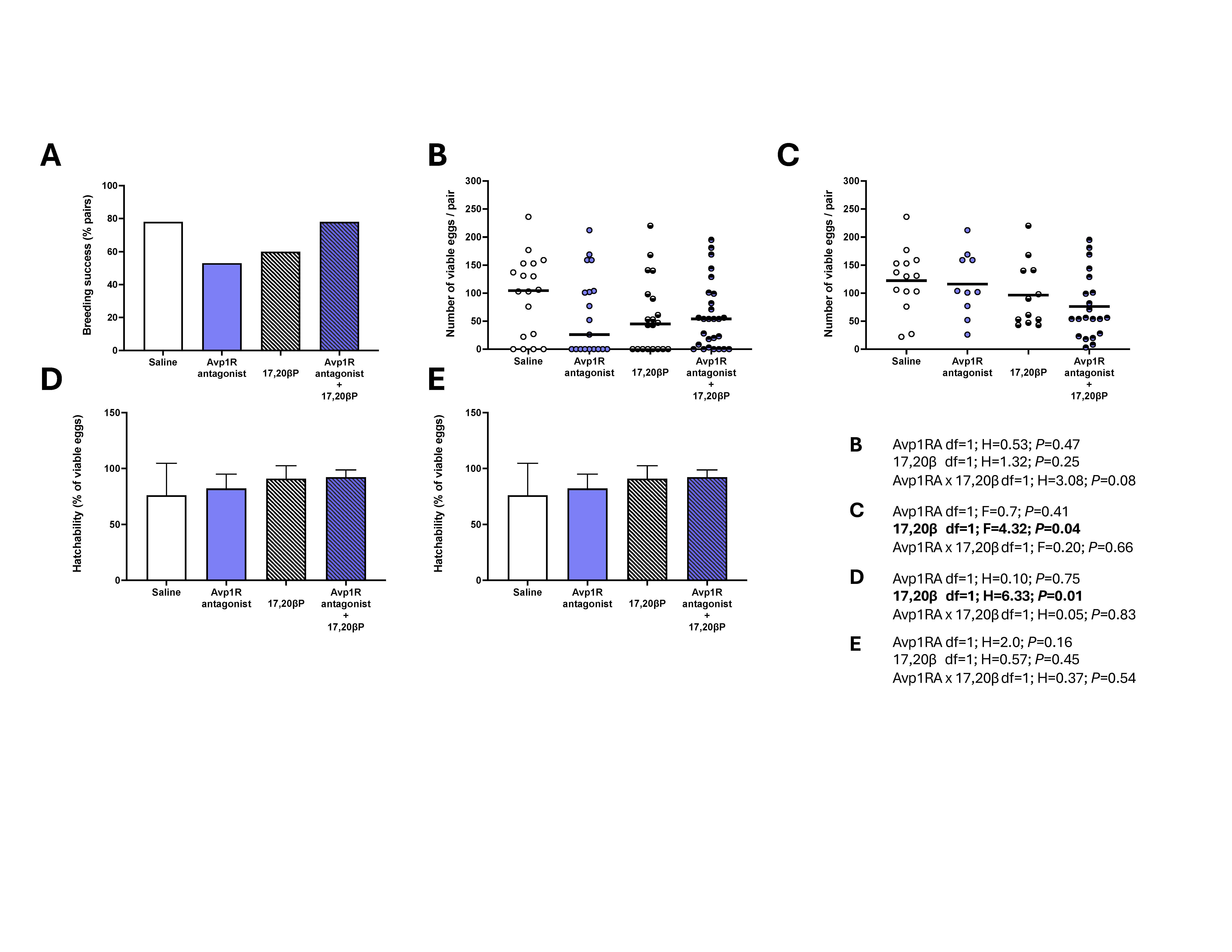

Supplement: SUPPLEMENTAL FILE S4 — Indices of reproductive success in breeding pairs following i.p. injection of physiological saline or 5 ng/g MC and EtoH vehicle or 60 nM 17,20bP, Breeding success in percent of all breeding pairs (A), median number of viable eggs produced (B), mean clutch size defined as mean number of eggs produced in successful breeding pairs (C), median hatchability as percentage of viable eggs from replicate fertilized egg batches (D), median 5 dpf survival as percentage of viable eggs from replicate experimental batches (E). Individual data points are indicated in addition to means and medians for viable egg count data. Parametric data were analyzed by two way-ANOVA, whereas non-parametric data were analyzed by two-way ANOVA on ranks with Scheirer-Ray-Hare extension. In cases of significant differences, post-hoc tests were used to resolve differences compared to saline control. Significant differences between treatment groups (P<0.05) are indicated by different letters. [file Image4.jpeg]

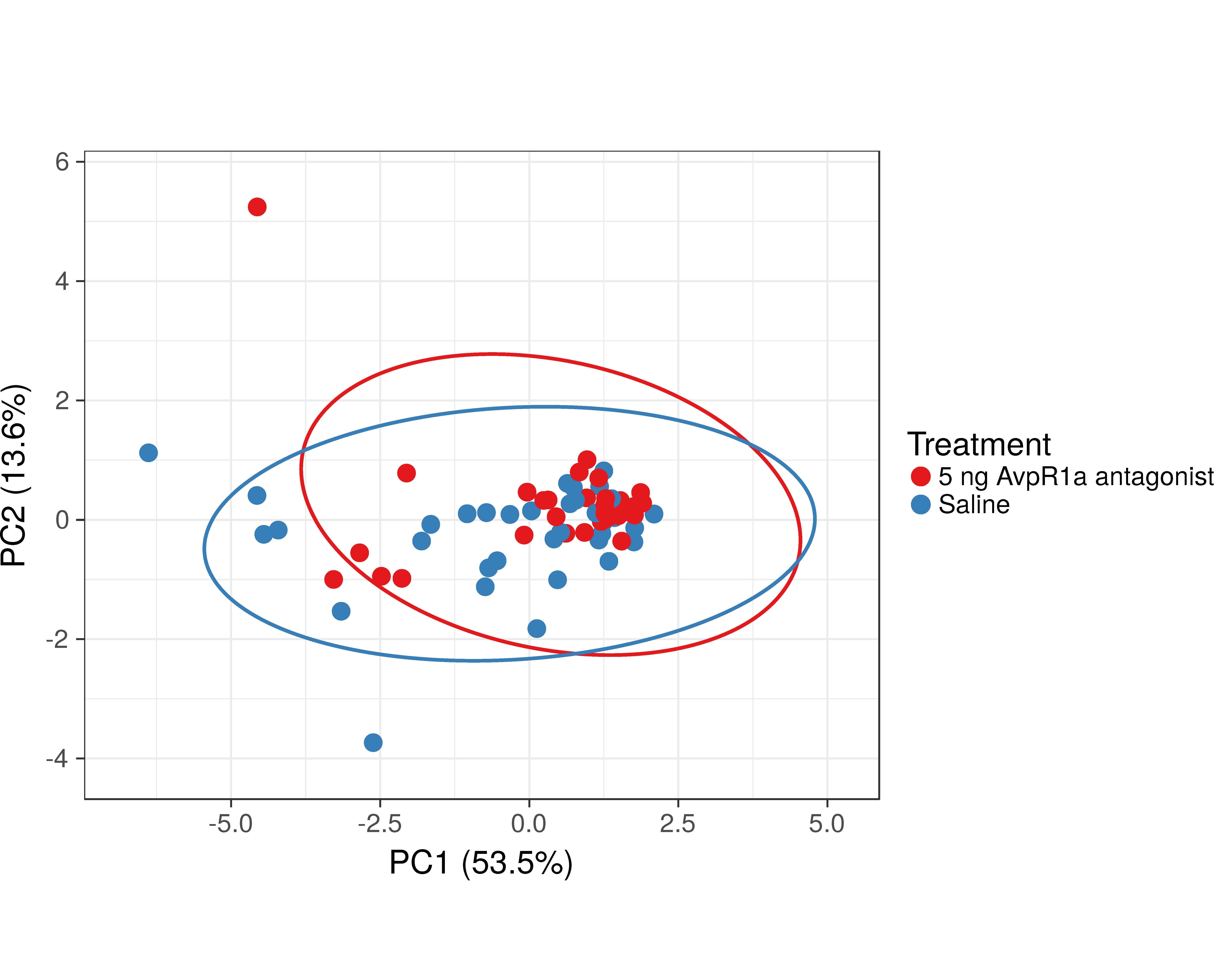

Supplement: SUPPLEMENTAL FILE S5 — Principal Component Analysis of all assessed courtship behaviours of physiological saline i.p.-injected and 5 ng/g i.p.-injected MC groups across experiments 1 and 2. Unit variance scaling is applied to rows; SVD with imputation is used to calculate principal components. X and Y axis show principal component 1 and principal component 2 that explain 53.5% and 13.6% of the total variance, respectively. Prediction ellipses are such that with probability 0.95, a new observation from the same group will fall inside the ellipse. n=67 datapoints. [file Image5.jpeg]
